# Lesson‐Drawing from New Zealand and Covid‐19: The Need for Anticipatory Policy Making

**DOI:** 10.1111/1467-923X.12893

**Published:** 2020-08-12

**Authors:** Sonia Mazey, Jeremy Richardson

**Keywords:** New Zealand, anticipatory, frenetic, expertise, leadership, implementation

## Abstract

The Covid‐19 pandemic has seen most governments worldwide having to think on their feet rather than implementing detailed and well‐rehearsed plans. This is notwithstanding the fact that a pandemic was bound to happen, sooner or later (and will happen again). The effectiveness of national responses has varied enormously. Globally, New Zealand has been perceived as setting the gold standard in ‘curve crushing’, and for a short period achieved Covid‐free status. For this achievement, much credit is due to the New Zealand government, especially to Prime Minister, Jacinda Ardern. However, post‐lockdown the New Zealand government has encountered a number of Covid policy implementation problems (many of which could have been anticipated). Nevertheless, Covid‐19 might still turn out to have been a seismic shock to existing policy processes and policy frames (such as austerity). If so, there are grounds for hope that in the future, governments and voters might be less short‐term in their outlook. Perhaps anticipatory, rather than reactive policy making, might become more fashionable?

## Introduction: welcome back the ‘nanny state’, all is forgiven?

our ‘new‐normal’ Covid‐19 world has generated a new, conventional wisdom, namely that ‘the economy will never be the same again’. We shall see. Certainly, some business sectors adapted quickly to online trading, and many workers and their employers discovered that working from home was possible and in some cases also more efficient. Thus, the pandemic proved to be a spark for business innovation and some of these changes will outlive the immediate crisis. Indeed, they will need to do so if a second wave of Covid‐19 sweeps the world, as seems possible. But, will government and politics ever be the same again? We must surely hope not, as the pandemic has highlighted vital and deeply worrying questions about the existing policy‐making capacity of governments around the world. Covid‐19 has severely tested the ability of governments to comprehend and respond effectively to complex policy problems, as well as their capacity to manage political agendas and mobilise public support for restrictive policies post‐lockdown.

The pandemic was a truly seismic shock to public policy‐making systems, as well as to firms, markets, and millions of employees. Most importantly, the pandemic has highlighted our collective dependence on government when the going gets really tough. Sadly, we have seen that when government is not up to the task many thousands of people die needlessly and economies edge close to collapse. Politicians (and, in practice, civil service bureaucracies) worldwide have become managers of a kind of mutual state insurance company, to whom poverty‐stricken individuals and massive multi‐billion pound companies alike turned for help as the pandemic took hold. Much research is underway by academic policy analysts evaluating how well different national governments fared; the evidence produced to date confirms that in most cases, government performance has not been impressive. We discuss some reasons for this generally poor performance below, but before doing so we turn to our adopted country, New Zealand, as it (and particularly its Prime Minister, Jacinda Ardern) has been crowned World Champion Covid‐19 Crusher.

## How did New Zealand do it?

Jacinda Ardern appears to have delivered US President, Donald Trump, a master class in crisis management. On the face of it, this is very odd. Ardern, at age thirty‐nine, is both young and relatively inexperienced in leadership. Before becoming Prime Minister in 2017, she had never run a big organisation and was said to have no burning desire to lead her country. Indeed, when she became leader of the Labour Party, her political opponents described her as ‘just a show pony’ and argued that ‘the stardust will soon fade’. By contrast, the seventy‐three year old US President had led the multibillion dollar Trump Organization since 1971 and had survived many business crises. Moreover, New Zealand was not well‐prepared for a pandemic, being ranked only thirty‐fifth out of 195 countries in the 2019 Global Health Security Index, with a poor overall score of only 54/100. Compare this with the US, which was ranked first out of 195 countries, with an overall score of 83.5/100. As one New Zealand academic health expert put it (bluntly) to the New Zealand Parliament’s Epidemic Response Committee, New Zealand was caught with its pants down when Covid‐19 struck. Similarly, another health expert has argued that the pandemic has exposed major shortcomings in the funding and delivery of public health services in New Zealand.

Notwithstanding the country’s unpreparedness for the pandemic and what some critics in New Zealand perceived to be an initially hesitant response, we should congratulate Ardern’s government on its handling of the coronavirus crisis. However, we must also acknowledge that this success owes more to effective fire‐fighting and strong crisis leadership than to *anticipatory policy making*, by which we mean governments conducting detailed advanced planning, and seeking detailed input from key interests likely to be impacted, *prior* to a possible major event. So, how did New Zealand achieve this remarkable success having been apparently not well prepared?

First and foremost, Ardern recognised that a global pandemic meant that specialist expertise was critical to effective policy making. The New Zealand/USA contrast in this regard is particularly instructive in terms of lesson‐drawing regarding crisis management. Contrast Ardern’s warm relationship with New Zealand Director General of Health, Dr Ashley Bloomfield, at the height of the crisis, with the virtual break down of relations between President Trump and Bloomfield’s US counterpart, Dr Anthony Fauci. In New Zealand, management of the Covid‐19 crisis was driven largely by appropriately qualified experts (mostly from within New Zealand, but also from overseas). Ardern and Bloomfield have stayed firmly on the same page and completely on message throughout the crisis. In contrast, Trump and Fauci seem to be not even reading from the same book. In summary, New Zealand appeared to be a textbook case of effective agenda management and the USA a textbook case of how to lose control of the agenda.

Secondly, Ardern came to this crisis with a lot of political capital. Her brief premiership has been characterised by the fact that even her strongest opponents like her. Whilst she is increasingly assertive in the rough and tumble of parliamentary debate and has started to exhibit some of the steel her critics had previously claimed she lacked, she remains a well‐liked and, above all, a *trusted* leader. Her inherent decency and humanity has been obvious to all, as demonstrated by her instinctive and heartfelt reactions to the massacre of fifty‐one Muslims at two mosques in New Zealand in 2019. In short, she rates high on trust and, particularly, on empathy, and very low on personal ego. She may not be a policy wonk (as we outline below, the government has struggled somewhat with the detailed, operational, policy implementation), but she demonstrated the crucial role of strong leadership and effective communication during a major crisis. Her Covid‐19 press conferences contained few references to ‘me’ and lots to ‘us’.

Thirdly, the ‘us’ is crucial in New Zealand. Ardern was careful to frame the government’s response as *our* response to the crisis, not hers, in great contrast to President Trump, for example. The emphasis was on national unity, with no blaming of her predecessors, opponents, or critics (always giving polite and usually smiling responses when pressed hard at her ‘must watch’ daily news conferences). Thus, ‘flattening the curve’ became a national challenge for New Zealanders. Having failed to make it to the final of the Rugby World Cup in 2019, Kiwis instead focussed their collective energies on winning the Covid‐19 Curve Crushing World Cup.

Thanks largely to this wave of public unity and support, the pandemic discourse quickly became about eliminating the virus (could we be the first in the world?), rather than managing it. Interestingly, the government’s initial response had been designed to limit the spread of the virus, so as not to overwhelm the seriously underfunded New Zealand health system. With fewer than 300 respirators available in the country at the start of the outbreak, the government’s primary objective was to slow down and hopefully contain the spread of the virus in order to limit pressure on the country’s limited health infrastructure. However, during lockdown, this objective morphed into making New Zealand Covid‐19 free. And when this goal was (temporarily) achieved, the Prime Minister publicly attributed this achievement to the efforts of the New Zealand ‘team of five million’. Owing to widespread public support for the ‘hard and fast’ lockdown, there were very few breaches of the rules (one notable exception being the Minister of Health). Everyday references to ‘Team New Zealand’ became common and it was a very effective mobilising device. New Zealand’s lockdown rules were very strict, but the ‘team of five million’ framing of the lockdown was an excellent example of how policy makers can ‘nudge’ citizens’ behaviour by resorting to simple, but effective messages.

Fourthly, New Zealand is both a unitary state and broadly speaking, a relatively united country. Unlike the US, Australian, and UK leaders, who have throughout this crisis had to ‘negotiate’ national Covid‐19 policy responses with influential, subnational governments, Ardern has not had to contend with the policy‐making implications and political divergences typically associated with devolved/federal polities. Similarly, while there are, of course, political cleavages and disagreements within New Zealand, there is no equivalent of the pro‐Trump *vs*. anti‐Trump divide in the US, or the visceral antagonism between the Remainers and Brexiteers in the UK. New Zealand’s pandemic response did not become entangled in bitter political warfare. Indeed, the then leader of the opposition was roundly condemned by the media and members of his own party for his criticisms (some of which turned out to be justified) of the government’s response. His alleged mishandling of the opposition’s response to the crisis also played a significant role in his subsequent ousting as leader of the opposition.

Fifthly (and most importantly), New Zealand was extremely lucky in that it was able to benefit from cross‐national learning. When driving in fog, keeping a close eye on the car in front is helpful. As the pandemic rolled around the world, New Zealand policy makers and the New Zealand public could see just how horrific things could get. The early Italian experience of Covid‐19 loomed large both in media coverage and in the minds of policy makers. The Italian case was a sharp reminder that some drastic domestic action was needed. Seeing the Fiat go off the road was, in effect, a very effective ‘spark’ leading to rapid policy change. By then, New Zealand policy makers had access to sufficient modelling data and medical expertise to know that a really severe lockdown was the only viable option if catastrophe was to be avoided. Given the limited capacity and fragility of its underfunded healthcare infrastructure, New Zealand could not afford, for example, to adopt Sweden’s controversial *laissez faire* approach to lockdown. Also, New Zealand business interests, though worried about the potential financial impact of the severe lockdown, did not mobilise to oppose it. Virtually everyone in New Zealand accepted that a severe lockdown was both necessary and inevitable. Countenancing a higher death rate than was absolutely necessary was in any case not in Ardern’s personal playbook. The decision to go ‘hard and fast’ was both a rational and a value‐based, *moral* decision for her. The severe, six‐week lockdown under alert Level 4 (possibly the most severe in any democracy), that followed in New Zealand, combined with a huge spending package to support employees and businesses, generated 87 per cent public support.

Glasses of New Zealand’s pinot noir all round then? A few perhaps, but not too many. In the event, successfully navigating the shift from severe lockdown to ‘open for business’ presented much tougher challenges for Ardern and her coalition government than leading the immediate response to the pandemic. Closing borders and placing New Zealand under severe lockdown was a simple and obvious decision to make. Whilst this decision certainly required courageous leadership in view of the huge risks to the economy, there were no other acceptable options. However, the policy‐making environment post‐lockdown has been much more complex, in several respects. For example, there has been considerable issue expansion (particularly concerns about the economy), a much wider range of interests have mobilised, and we have witnessed several examples of detailed policy implementation problems that could have been anticipated (and therefore avoided). As a consequence, the post‐lockdown period has proved to be much more challenging for the government.

## **How high hopes in Wellington were dashed in hotels in Auckland** ^1^

Post‐lockdown Covid‐19 policy making in New Zealand quickly started to encounter some very basic implementation problems. The New Zealand Covid‐19 story is a game of two halves: strong defence and no own goals in the first half, but plenty of defence errors and own goals after the half‐time break. Closing New Zealand’s international borders before most other countries (on the advice of epidemiologists) and instigating a severe lockdown relatively early, now looks like the simple ‘no brainer’ decision that it was. By contrast, moving out of lockdown (Level 4) to the current Level 1 (essentially a return to normal life, but with very restrictive border controls), has highlighted the absence of effective anticipatory policy making prior to the pandemic.

First, there was confusion over what moving from Level 4 to Level 3 would mean in practice—for example, how would primary schools operate social distancing? On this particular issue the education sector complained it had not been appropriately consulted prior to decisions being made. However, it is the management of the very strict border controls and the associated quarantine processes that gave rise to classic policy implementation problems. These problems have threatened to undermine the government (and Jacinda Ardern’s) reputation as a safe pair of hands during the pandemic, and have given the government’s critics some open goals to shoot at. The political discourse shifted from ‘Team New Zealand’ to ‘shambolic’ quite quickly as the country moved from alert Level 4 lockdown. As always with big policy decisions, the devil is in the detail.

In fact, Ardern had repeatedly warned that New Zealand would not stay Covid‐19 free indefinitely so long as the pandemic continued to worsen worldwide. The government had early on rejected advice from some epidemiologists that New Zealand should not allow its own citizens to return (as some other countries did). Thus, it was inevitable that as international flights resumed and New Zealand citizens and permanent residents started to return home, new cases of Covid‐19 would be imported into the country. In preparation for these new cases, the government had introduced government funded, mandatory fourteen‐day quarantine in designated hotels in Auckland and Christchurch. The Director of Health, Ashley Bloomfield, instructed officials that all those in quarantine should be tested on day three and day twelve of their fourteen‐day isolation. However, the old adage with which academic public policy analysts are very familiar, namely, ‘he/she who implements, decides’ came into play. What was supposed to happen in the quarantine hotels did not, and for a period the government was forced to act swiftly to plug gaps in the quarantine and tracing systems, amid a media frenzy suggesting that things were falling apart.

The most notable example (initially uncovered by the media), was the early release of two women from quarantine on compassionate grounds without prior testing, who were allowed to travel unsupervised to their destination. To make matters worse, they got lost and came into close contact with friends who helped them to get back *en route*. More cases continued to come to light, until the government was forced to acknowledge that, in fact, several hundred people had been released from the quarantine hotels without being tested. Tracing these people, as well as all of their contacts proved difficult, not least because it quickly transpired that many of them had provided false telephone numbers on leaving quarantine. Having spectacularly eliminated community transmission of the virus, the government subsequently failed to put in place effective quarantine measures for people entering the country. Other basic quarantine management errors that came to light included allowing new arrivals to mix with those who had been in quarantine for many days, and even allowing some in quarantine to go for a stroll in an Auckland street, rubbing shoulders with passers‐by. Further problems arose over lax security measures at the quarantine hotels (some ‘inmates’ escaped, though were quickly returned), and serious questions are now being asked about the legality of forcing New Zealand citizens and permanent residents returning to New Zealand to remain isolated for fourteen days in a hotel room. Leaving aside this issue, the government is now grappling with two key problems: managing the *flow* of returnees (estimates vary, but as many as 1 million Kiwis are thought to live overseas); and deciding who should pay for these returnees’ mandatory quarantine—the taxpayer or the returnees? Both issues are becoming increasingly politically salient.

To be fair, the government has been effective at both ‘re‐steering’ the implementation process as each problem has arisen. It has also drafted in more able ministers to fix problems as they have arisen. The hapless Minister of Health, David Clark, was finally replaced by the extremely competent Education Minister, Chris Hipkins, and the government's ‘go to’ troubleshooting Housing Minister, Megan Woods, was placed in charge of the quarantine system. On appointment, Woods immediately set up a three person ‘review of managed isolation and quarantine’, which carried out the review over just two days in late June. The review found that the system was, indeed, ‘under stress’ (but not broken), and proposed a raft of detailed recommendations for improvement. In short, the overriding tone of the review team’s report is ‘let’s get a firm grip on this’.^2^ In introducing the report, Woods argued that ‘there is no playbook for this kind of pandemic’.^3^ However, the report, whilst demonstrating that no playbook seemed to exist, actually shows that something akin to a playbook could have been ready well in advance of a pandemic. Many, if not most, of the detailed implementation problems that emerged could have been avoided by careful and detailed planning in advance of any pandemic, that is, by the type of anticipatory policy making that we are advocating here. The absence of such plans is something for which all previous New Zealand governments must take some blame and this failure did place at serious risk the huge success that the tough lockdown had achieved.

However, as outlined above, as these problems arose, the government demonstrated the same quality as when the pandemic first broke out, namely an ability to think on its feet and to mobilise the necessary expertise. The government quickly ended quarantine release on compassionate grounds; it deployed the New Zealand Defence Force (NZDF) to manage the quarantine process (some 168 NZDF staff were deployed across twenty‐seven quarantine facilities); and it ratcheted up testing of border workers, staff at quarantine facilities, and international airline crews. So, whilst the government had not been anticipatory enough, it proved quite resilient (see below) when things went wrong. This ongoing re‐steering of the implementation process seems to be working, so much so that one media outlet reported that the situation in one of the Auckland hotels had changed from ‘Fawlty Towers’ to ‘a well‐run ship’. That said, in mid‐July, the fine tuning of the implementation process continued to be work in progress. As a consequence, New Zealand remained vulnerable to a second wave of Covid‐19, in part because it did not yet have an effective contact tracing system in place. At that time, only one in eight New Zealanders had downloaded the government’s contact tracing app and of those that have, only one in sixty actually scans a single bar code per day. Here, perhaps, we see the biggest problem facing all governments in dealing with the pandemic, namely how to change the *behaviour* of citizens. Though there are no known instances of ‘Covid parties’ in New Zealand, ‘Team New Zealand’ has become very relaxed indeed under Level 1, leading to pleas from both Ardern and Ashley Bloomfield for everyone to download and use the app. The pleas have been backed up with warnings that lockdowns of some kind might need to be re‐introduced.

So, what does the New Zealand case tell us? Four lessons stand out. First, situations like the pandemic demand that politicians rely on experts and refrain from second guessing people who know a lot more than they do. Second, politicians need the courage to make very tough (and often unpopular) decisions in order to manage a crisis situation. In New Zealand’s case the economic consequences of Ardern’s courageous leadership will no doubt be very high. A particular example is the tourist industry, which is New Zealand’s biggest export industry, contributing 21 per cent of foreign exchange earnings and representing 5.8 per cent of GDP, with some 8.4 per cent of the workforce employed in the industry (as at 31 March 2019).^4^ Closed borders are little short of a disaster for the industry. Third, politicians need political skills to ‘sell’ tough decisions. Prime Minister Ardern and her Finance Minister, Grant Robertson, performed superbly in this regard, as did Ashley Bloomfield, who became something of a cult figure during the height of the crisis. Indeed, the fact that the then Health Minister appeared to blame Bloomfield publicly for some of the implementation problems outlined above, virtually ensured that the Minister finally had to go. Fourth, New Zealand, as we have suggested, got lucky by being able to watch the car in front in the Covid‐19 fog. However, relying on a luck in crises is not a good idea. Thus, we now turn to a discussion of how governments might avoid having to rely so much on luck and thinking fast on their feet, not just with regard to pandemics, but with respect to a raft of serious policy problems, including those currently staring us all in the face, such as global warming, and rapidly increasing obesity rates.

## Office retention or a good obituary: can politicians be more anticipatory in their policy making?

The main lesson to be drawn from this crisis is that all governments, including Covid‐19 podium countries like New Zealand, need to be much more *anticipatory* rather than reactive in their approach to public policy making.^5^ Dreaded events might never happen, but when they do the resulting disruption is massive. Unfortunately, rather than anticipating problems ahead and devoting time and resources to avoid them occurring, or mitigating the impact of such an event (such as a pandemic), many governments have for decades been travelling in the opposite direction. Britain is a case in point. As King and Crewe suggest, ‘in previous generations, foreign observers of British politics viewed the British political system with something like awe. Government in Britain was not only highly democratic: *it was astonishingly competent. It combined effectiveness with efficiency*’.^6^ However, they go on to observe: ‘sadly, the British system is no longer held up as a model, and we suspect that one reason is that today’s British governments screw up so often’.^7^ King and Crewe’s seminal volume is replete with examples of British governments screwing up. Of particular relevance to our pleas for a more *anticipatory* policy style is their finding that ‘there is at the heart of the British system a lack of *deliberation*’.^8^ Deliberation implies a measured (and generally slow, as opposed to frenetic), approach to policy making. It also implies the mobilisation of expertise, including that possessed by interest groups who know what will work on the ground and what will not. Thus, the British government’s stumbling (at least from a New Zealand perspective) response to Covid‐19, hard on the heels of the policy‐making ‘omnishambles’ of the UK’s departure from the EU, is nothing unusual.^9^

Alas, Britain is not alone in exhibiting a broken policy‐making system. Sweden was long thought to be the model of a rational, future‐focussed, anticipatory policy planning, particularly via its emphasis on the mobilisation of interest group expertise and the use of policy commissions. However, as Petersson argues, ‘neo‐corporatist arrangements, such as interest groups being represented in the board of state agencies, were dismantled back in the 1990s’ and ‘commissions of inquiry have more or less ceased to be an arena for negotiation and consensus seeking’.^10^ Interestingly, Petersson also argues that the *pace* of policy making in Sweden has changed. He argues that commissions are now given much less time to complete their work and that ‘this change from formal and long‐term to informal and short‐term procedures means, together, that the Swedish commissions of inquiry have lost their unique function in the production of a qualified knowledge base for political decision‐making’.^11^

The increasingly *frenetic* pace of public policy making, together with the diminution (sometimes sheer denigration) of *expertise* seems to be a well‐established trend in western democracies, exacerbated by the rise of populism. Populism and rational, expert led, *deliberative* policy making sit uneasily alongside each other. Indeed, Jennings et al.’s typology of policy blunders includes what they term ‘hyper‐excited politics’ whereby ‘politicians and officials rush policy announcements only to repent at leisure when these commitments turn out to be counter‐productive, more costly than expected, fail to achieve intended outcomes, or generate no interest’.^12^ They go on to suggest that ‘“fast thinking” by key decision makers acting under political pressure may make them vulnerable to cognitive biases, such as disproportionately “locking onto” particular bits of information’.^13^ In similar vein, David Halpern,former head of Number Ten’s Behavioural Insights Team, describes life behind the shiny black door of Number Ten as akin to a hospital accident and emergency department.^14^ He comments that ‘in such a world, there’s often not the time, nor the patience, for the answer to be “more research needed”, let alone a randomised control trial—though perhaps there should be’.^15^ Moran has also highlighted a similar phenomenon as one of the possible causes of policy disasters via the extension of the regulatory state. ‘Macho management, hiving off expertise and experts from the point where strategic decisions are made; cutting personnel in pursuit of leaner forms of organisation: all increase the likelihood that *strategic decision‐making will be hasty, unsupported by evidence* and made without the support of those whose co‐operation is needed for effective implementation’.^16^ The lack of time and resources for proper analysis appears to be a common phenomenon. For example, Van Nispen and Scholton note that ‘the role of policy analysis in inducing learning in times of crisis seems rather limited’, and that ‘policy change seems to be primarily due to powering; puzzling takes place mainly in the shadow of powering’.^17^

These characterisations of modern‐day policy making are deeply worrying, but introducing an ‘anticipatory policy style’ will be difficult to achieve. Governments come and go at relatively short intervals (potentially, every three years in New Zealand). Implementing effective and often expensive policies in preparation for an event that may or may not occur at some unknown future date brings few electoral benefits for a sitting government. Anticipatory policy making is arguably a mug’s game for any government: the costs are immediate, but the political rewards of foresight are uncertain and likely to benefit a successor government, years into the future. Politicians are more worried about office retention than receiving a laudatory obituary acknowledging their policy foresight when they were in office. Healy and Malhonta’s study of preparedness for natural disasters provides some depressing clues as to why anticipatory policy making is not very common. Their findings suggest that governments will under‐invest in natural disaster preparedness. As they explain:

Voters are, in a word, myopic … they are myopic in the sense that they are unwilling to spend on natural disasters before the disasters have occurred. An ounce of prevention would be far more efficient than a pound of cure, but voters seem interested only in the cure. The resulting inconsistencies in democratic accountability reduce public welfare by discouraging re‐election‐minded politicians from investing in protection, while encouraging them to provide assistance after harm has already occurred.^18^

Whilst there appear to be relatively few political incentives for politicians to introduce anticipatory policies, it is probable that there are even fewer politicians of stature, who have the courage to explain to electorates some of the realities of the world and to make tough, unpopular, decisions. The consequences of global warming, if left unchecked, will dwarf Covid‐19 as a crisis, but neither elected politicians, nor most voters, seem to care. The explanation for this is simple: ‘jam tomorrow’ is not a strong selling point to the average voter.

However, might the Covid‐19 crisis make anticipatory public policy making more likely, precisely because it has so clearly illustrated the kind of train wreck caused by a lack of anticipatory policy making? The Covid‐19 train wreck has not just cost hundreds of thousands of lives, it has wreaked havoc with economies around the globe and impacted individual workers and huge multinational companies alike. There might be grounds for hope. Covid‐19 might possibly turn out to have been a seismic event in the process by which public policies are made.

First, Covid‐19 has demonstrated beyond doubt that when the going gets tough, the public relies on governments, not markets, to come to the rescue. New Zealand has survived the crisis thus far very well because government has ‘taken charge’. Big government is back with a bang (and very big bucks).

Second, popular support for public welfare provision has resurged globally. Like the rest of the world, New Zealand is experiencing an outbreak of ‘grantitis’. Almost anything that moves seems to be eligible for a government grant or subsidy. New Zealand Finance Minister, Grant Robertson, is both Grant by name and grant by nature! Indeed, the elephant in the room, post‐Covid, will almost certainly be the costs and consequences of hastily drafted economic rescue packages. Grantitis has stopped in its tracks the austerity ‘policy fashion’ that itself spread like a virus for decades around the world. As Blyth has argued, as an idea ‘austerity has been astonishingly successful and serves as the contemporary instruction sheet’.^19^ However, the mantra that ‘there is no money’ no longer rings true. It has taken one virus to kill another. Fiscal constraint, prudence, and ‘good housekeeping’ rules that since the 1980s framed and severely constrained debates about what is or is not possible across almost every policy sector (and across national boundaries) now look very outdated.

Third, *expertise* is back in fashion. As we have suggested above, the New Zealand response to the pandemic was, essentially expert and evidence driven. There was no hint from Ardern that she might have read somewhere on the internet of a magic cure. To be sure, there were ‐ and always will be differing views amongst experts and it is the unenviable job of politicians to make policy decisions based on conflicting and/or partial expert advice. ‘Experts’ are themselves interested parties, often belong to rival epistemic communities, and, of course, can be badly wrong. The contrast between the New Zealand and the Swedish pandemic response, to which we drew attention above, is illustrative in this regard. Both political responses were expert driven, but have resulted in very different policy trajectories. Time will tell which of these two policy responses was more effective in the longer term in terms of saving lives *vs*. minimising economic impact. We should not shy away from this reality. Politicians are tasked to make tough decisions based on their own values and principles, as well as expert advice. We live in a democracy not a technocracy. As highlighted above, the decision by the New Zealand government to allow its citizens and permanent residents to return home was an ethical and political decision taken in full knowledge of the concomitant health risks to the public. Similarly, the government rejected Dr Bloomfield’s recommendation that New Zealand remain at alert Level 2 for a significantly longer period than the government eventually decided. This divergence reflected the gradual expansion of the Covid‐19 policy‐making agenda and increasing interest group mobilisation. Initially the government was focussed on saving lives, but as time passed, it also had to respond to increasing concerns about economic recovery. Business interests, which had initially supported the lockdown, became increasingly vocal about the need to open up the economy. With the general election just weeks away (on 19 September) the government also came under increasing political pressure from its coalition partner, New Zealand First, a minor party keen to distance itself from the Labour Party, as well as from the pro‐Business opposition National Party. Inevitably, ‘politics as normal’ began to emerge once the curve had been crushed. No doubt this will always be the case, but relying on evidence and expertise as a starting point for decision making, rather than political ideology or gut feelings, or following populist demands, is, essential if future policy disasters are to be avoided.

## Conclusion

The overriding lesson of Covid‐19 is that societies—not just governments—need to be more *anticipatory*. Anticipatory policy making is expensive and time consuming (even boring perhaps as it is often about detail, rather than big bang decisions).^20^ Nevertheless, for known major risks it is likely to be a good long‐term investment. Though the New Zealand response to the pandemic has thus far been effective, the government could still have done better in terms of anticipatory policy making. However, for many other countries Covid‐19 has been a policy disaster with huge human consequences. This pandemic is a massive, global, wake‐up call for both governments and voters, who will surely face more challenging transnational crises. We are not, of course, arguing that anticipatory policy making is easy, cheap, or that governments can always avoid crises via anticipatory policy making. As we have suggested, it requires a lot of deliberation, which is time consuming and costly. Also, anticipating what needs to be done in a particular crisis, such as a global pandemic, is not a one‐off exercise. The policy plan needs periodic and detailed revisiting. For example, the fact that the stockpile of masks in the UK was past its use‐by date does not mean that anticipatory policy making does not work. Rather, it suggests that the stockpiling system should have been designed to give an alert that it was time to replace the masks with a new batch.^21^

However, there will be many crises that unlike Covid‐19 could never have been predicted, or even if predicted, simply pan out in unpredictable ways. Anticipatory policy making, as Wildavsky argued in his seminal work *Searching for Safety*, will not *per se* always be the solution—though this is not to say that nothing can be done in advance to lessen the serious effects of the crisis.^22^ Additionally, as in the New Zealand government’s response to the pandemic, ‘resilience’ as a strategy often comes in to play:

A strategy of resilience … requires reliance on experience with adverse consequences once they occur in order to develop a capacity to learn from the harm and bounce back. Resilience, therefore, requires the accumulation of large amounts of generalizable resources—such as organizational capacity, knowledge, wealth, energy, and communication—that can be used to craft solutions to problems that the people involved did not know would occur.^23^

Finally, we need to recognise that ‘the government’ has borrowed huge amounts of money in most affected countries in order to get through this crisis. However, ‘the government’ is not a separate entity from society. As citizens, we now have a *collective* debt. Meeting this liability will require controversial policy changes in very many sectors, and much courage from our politicians. Policies previously ruled out as being too unpopular, such as higher taxes, extending the tax base, or raising the retirement age, will undoubtedly come back onto the political agenda. It will take decades to pay off these massive debts. Clearly, the pandemic has demonstrated that money can be found at the drop of a hat, but, alas, governments do not have orchards of money trees. We were told that there would be a pandemic one day. In hindsight, we should all have paid a lot more attention to the experts who warned us, very clearly, that this would happen.

**Note**: An earlier version of this article was published in the COVID‐DEM Infohub.

## Notes

^1^
We borrow from the title of the seminal work on implementation, J. Pressman and A. Wildavsky, Implementation. How Great Expectations in Washington Are Dashed in Oakland; Or Why It's Amazing that Federal Programs Work at All, Berkeley, University of California Press, 1973.

^2^
See New Zealand Government, Review of Managed Isolation and Quarantine, 28 June 2020; https://covid19.govt.nz/updates‐and‐resources/latest‐updates/miq‐review/ (accessed 22 July 2020).

^3^
New Zealand Government, ‘Government strengthens managed isolation system’, 28 June 2020; https://www.beehive.govt.nz/release/government‐strengthens‐managed‐isolation‐system (accessed 22 July 2020).

^4^
Tourism New Zealand, ‘About the tourism industry’, 27 May 2020; https://www.tourismnewzealand.com/about/about‐the‐tourism‐industy/ (accessed 27 July 2020).

^5^
For a discussion of different policy styles see, J. Richardson, British Policy‐Making and the Need for a Post‐Brexit Policy Style, London, Palgrave Macmillan, 2018.

^6^
I. Crewe and A. King, The Blunders of Our Governments, London, Oneworld, 2013, p. ix, emphasis added.

^7^
Ibid.

^8^
Ibid., p. 652, emphasis added.

^9^
For a discussion of long‐term changes in the British policy style, see J. Richardson, ‘The changing British policy style: from governance to government?’, British Politics, vol. 13, no. 2, pp. 215–233; and for a discussion of the Brexit saga as an omnishambles, see J. Richardson and B. Rittberger, ‘Brexit: simply an omnishambles or a major policy fiasco?’, Journal of European Public Policy, vol. 27, no. 5, 2020, pp. 649–65.

^10^
P. Petersson, ‘Rational politics, commissions of inquiry and the referral system in Sweden’, in J. Pierre, ed., The Oxford Handbook of Swedish Politics, Oxford, Oxford University Press, 2015, p. 653.

^11^
Ibid.

^12^
W. Jennings, M. Lodge and M. Ryan, ‘Comparing blunders in government’, European Journal of Political Research, vol. 57, no. 1, 2018, pp. 238–258.

^13^
Ibid.

^14^
D. Halpern, Inside the Nudge Unit. How Small Changes Can Make a Big Difference, London, W. H. Allen, 2015, p. 185.

^15^
Ibid., p. 186.

^16^
M. Moran, ‘Not steering but drowning: policy catastrophes and the regulatory state’, The Political Quarterly, vol. 72, no. 4, 2001, pp. 414–427, at p. 415, emphasis added.

^17^
F. K. M. Van Nispen and P. W. A. Scholten, ‘Policy analysis in times of austerity: puzzling in the shadow of powering?’, Journal of Comparative Policy Analysis, 2015, pp. 439–447.

^18^
A. Healy and N. Malhotra, ‘Myopic voters and natural disaster policy’, American Political Science Review, vol. 103, no. 3, 2009, pp. 387–406.

^19^
M. Blyth, Austerity: The History of a Dangerous Idea, New York, Oxford University Press, 2013, p. 176.

^20^
For a discussion of the obstacles to anticipatory policy making see R. DeLeo, Anticipatory Policymaking. When Government Acts to Prevent Problems and Why It Is So Difficult, Abingdon, Routledge, 2016.

^21^
Both our cars tell us, without human intervention, when a service is due. More importantly, for us here in NZ, writing this article has reminded us that the contents of our ‘earthquake shed’ in the garden need updating as the emergency supply of food and water is beyond the use by date. Thus, we have been very ‘anticipatory’ but have no system to remind us that we need to regularly replace supplies. Back to the drawing board there then!

^22^
A. Wildavsky, Searching for Safety, New Bruswick, Transaction Books, 1988.

^23^
A. Wildavsky and A. Wildavsky, ‘Risk and safety’, The Library of Economics and Liberty, not dated; https://www.econlib.org/library/Enc/RiskandSafety.html (accessed 15 July 2020).

